# Use of processing fluid samples for longitudinal monitoring of PRRS virus in herds undergoing virus elimination

**DOI:** 10.1186/s40813-019-0125-x

**Published:** 2019-08-01

**Authors:** Giovani Trevisan, Eva Jablonski, Jose Angulo, Will A. Lopez, Daniel C. L. Linhares

**Affiliations:** 1 Veterinary Diagnostic and Production Animal Medicine, Iowa State University, Ames, Iowa USA; 2 Zoetis, Parsippany, New Jersey USA

**Keywords:** Porcine reproductive and respiratory syndrome virus, Processing fluid, Time to stability, Herd closure

## Abstract

This was an observational study that prospectively followed 29 breeding herds for 65 weeks in the U.S.A. that became infected with porcine reproductive and respiratory syndrome virus (PRRSv). The herds operated in a four-week batch farrowing system and adopted a load-close-expose strategy using a modified-live virus vaccine to achieve PRRSv stability. The purpose of this study was to describe time to stability (TTS) based on RT-qPCR testing for PRRSv RNA on processing fluid samples in herds undergoing PRRSv elimination, after implementing herd closure and mass exposure to a PRRS modified-live virus (MLV) vaccine. For the purpose of this study, stability was defined as consistently producing PRRSv-negative pigs. Study herds were monitored until two consecutive piglet batches tested PRRSv RT-qPCR negative, then 30 due-to-wean piglet sera from the second batch were tested for PRRSv RNA by RT-qPCR. Once the farm re-opened, sera from incoming naïve gilts were tested for anti-PRRSv antibodies by ELISA at 30- and 60-days post-entry to confirm negative status to PRRSv. Day zero was the day of whole-herd exposure to a commercial PRRS vaccine virus. Twenty-eight of 29 herds (96.55%) achieved TTS within the study period. TTS ranged from 18 to 55 weeks with a median of 27 weeks. Serum from due-to-wean piglets was collected on 28 farms, of which 26 (92.85%) obtained PRRSv RT-qPCR-negative results on the first collection. At the end of the observational period, 16 sow farms successfully re-introduced PRRSv-naïve gilts with no detected serologic response. In conclusion, the median time to achieve TTS in breeding herds being operated in a four-week batch farrowing system undergoing PRRSv elimination using load-close-expose with attenuated virus vaccine was 27 weeks. Also, processing fluid-based monitoring of breeding herds under PRRS elimination was practical and reliable to assess PRRSv stability.

## Introduction

The economic impact of porcine reproductive and respiratory syndrome virus (PRRSv) in USA breeding herds was estimated to be $302.06 million per year [1]. Understanding when infected breeding herds start producing PRRSv-negative pigs is crucial to make vaccination and other health-related decisions for disease control in growing pigs.

The current American Association of Swine Veterinarians (AASV) guidelines for monitoring PRRSv in breeding herds undergoing elimination consist of obtaining serum samples from due-to-wean piglets [2]. Herds are considered ‘stable’ when four consecutive monthly negative results of 30 piglets are achieved, tested in pools of 1:5 by PRRSv RNA by RT-qPCR. This strategy assumes that PRRSv infection dies out within 90 days after reaching prevalence under 10%. However, field studies have suggested that 30 monthly samples from due-to-wean piglets lack sensitivity to detect PRRSv in low prevalence scenario [3–5], suggesting the need for more sensitive PRRSv monitoring schemes, for instance by surveying more pigs, more frequently.

In that regard, a procedure using processing fluids to monitor PRRSv in three-to-five-day-old piglets has been described [6–8]. Processing fluid is described as the serosanguinous fluid recovered at the time of castration and tail docking [6]. The probability of PRRSv RNA detection by RT-qPCR was greater when using 1 aggregated processing fluid sample from all pigs submitted to castration and tail docking compared to that of using 30 piglet serum samples [6]. Processing fluids are a more convenient sample type that allow for more litters and more pigs to be sampled across farrowing rooms, while reducing overall diagnostic cost as compared to multiple individual pig serum samples. To our knowledge, the pattern of PRRSv RNA detection in processing fluids over time in herds undergoing PRRSv elimination is still unknown. The purpose of this study was to describe time to stability (TTS) based on RT-qPCR testing for PRRSv RNA on processing fluid samples in herds undergoing PRRSv elimination, after implementing herd closure and mass exposure to a PRRS modified-live virus (MLV) vaccine.

## Material and methods

### Overview of the study design

This was a prospective study conducted on 29 commercial sow farms, which were infected with PRRSv and adopted a herd closure program to eliminate the virus without depopulation. Herds were monitored for PRRSv RNA by testing processing fluids using PCR-based methods. PRRS shedding status of each herd was then further confirmed with blood testing of due-to-wean piglets, and by ELISA from incoming naïve gilts. This study was approved by the Iowa State University Institutional Animal Care and Use Committee under protocol number 3-18-8730-S.

### Study herds, and PRRS elimination program

The study recruited PRRSv-naïve (i.e. AASV category four) [2] breed-to-wean herds that became infected with PRRSv by the introduction of shedding gilts, and adopted a load-close-expose program to eliminate the virus without depopulation. The sow inventory of the farms operated in batch-farrowing system ranged from 422 to 859 sows (median 544), and the sow inventory of farms operated in continuous breeding system ranged from 2,392 to 2,434 (median 2,413). For this study, load-close-expose was defined as implementation of a herd closure program (i.e. temporary interruption of replacement gilt introduction until there was sufficient evidence of lack of PRRSv shedding in the population), along with vaccination of all breeding-age pigs with one dose of Fostera® PRRS vaccine (Zoetis, Parsippany, NJ, USA) within 15 days of PRRSv detection. All study herds were vaccinated between March and October 2017. Piglet weaning was performed weekly on two farms and every four weeks in a batch-farrowing program for the remaining farms (*n* = 27). For the farms operating in the batch farrowing system, all batches of pigs were weaned at the same day. On the farms operated on continuous flow, a weekly flow was implemented, following all in / all out procedures at room level by week. Day zero was the day of MLV vaccine exposure.

### PRRS monitoring, and diagnostic testing

The collection of processing-fluid samples started at 9 weeks post MLV vaccination, and continued every four weeks for a period of 59 weeks post-MLV, or until processing fluids tested negative for PRRSv RNA by RT-qPCR on two consecutive batches. Processing fluids collection was conducted during castration and tail docking procedure (i.e piglet processing time), following guidelines by Lopez et al [6]. For batch-farrowing farms, processing fluids were obtained at all piglet processing times, every four weeks, starting at nine weeks post-MLV vaccination. One aggregated processing fluid sample per farrowing room per day was collected and stored at − 20 °C. The daily obtained fluids were stored in 50 mL Falcon Tubes (Fisher Scientific, Waltham, MA), frozen at -20 °C, and submitted to the Iowa State University Veterinary Diagnostic Laboratory (ISU-VDL) for testing with the use of commercial kits. Processing fluids over multiple farrowing rooms and multiple days were pooled into 1 weekly sample by the ISU-VDL personnel, representing approximately 90% of all litters with processed pigs (i.e. pigs submitted to castration and tail docking). The range of piglets contributing for a pooling sample tested per farm per week was from 700 to 2,100 piglets. Samples were tested by PRRS RT-qPCR using the commercial Applied Biosystems TaqMan® kit for North American and European PRRSv RNA. The results were reported as quantification cycle (Cq), formerly known as Cycle Threshold (Ct) values by the ISU-VDL. As instructed by the PCR kit manufacturer and the ISU-VDL, Cq values equal or above 37 were considered negative. After the second consecutive negative RT-qPCR result on processing fluids, 60 blood samples were taken from the same piglet cohort, within two days of weaning, and also tested by PRRSv RT-qPCR at the ISU-VDL using the same commercial PCR reaction previously described. After achieving two consecutive negative RT-qPCR results for PRRSv RNA detection on processing fluid, and one negative result at weaning-age using 60 blood samples, incoming gilts were introduced to the breeding herds. Blood samples were collected from gilts within 30, and subsequently at 60 days after herd introduction.

Serum samples from gilts were tested at the ISU-VDL by IDEXX PRRS X3 Ab ELISA. Gilts were considered seronegative when sample to positive (S/P) ratio was below 0.400, as instructed by the kit manufacturer.

### Outcomes

The major outcome of interest in this study was time to achieve two consecutive negative results (time to stability = TTS) of PRRSv RNA testing by RT-qPCR on processing fluids samples, followed by PRRSv RT-qPCR -negative results on due-to-wean piglet sera testing, and ELISA-negative results on incoming gilt sera. TTS was described with survival analysis (Kaplan-Meier) using PROC Lifetest from SAS 9.4 (SAS Institute Inc., Cary, NC). Dropped farms were censored at the respective time after MLV vaccination. Also, farms were censored if not reaching the event (TTS) by 60 weeks after day zero. Descriptive statistics was used to report the frequency of PRRSv RNA detection by RT-qPCR on sera collected from weaning-age piglets after reaching two consecutive negative results on processing fluids. Similarly, the pattern of ELISA results of incoming gilts was described. Reported confidence intervals (CI) for proportions were calculated assuming binomial proportion distribution with a Wald approximation.

## Results

**Fig. 1:**
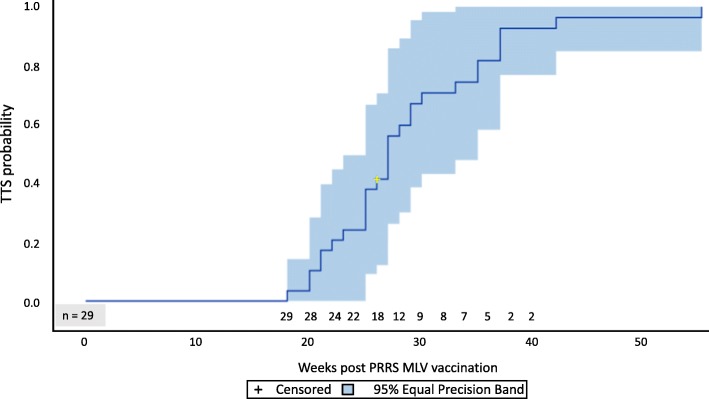
Time in weeks to achieve two consecutive negative results for PRRSv by RT-qPCR using processing fluids after herd exposure to MLV. Kaplan–Meier survival curve estimates for time to obtain two consecutive PRRS PCR-negative test on processing fluids (TTS). Cross marks represent censored data. The letter ‘n’ represents the number of study herds at risk at each point in time

Of the 29 total herds enrolled in this study, one farm dropped out before concluding all phases of the study, and another two right after achieving TTS, due to discontinued production. Among all enrolled herds, 28 (96.55%, CI 89.91 to 100%) achieved TTS within the 59 weeks of follow-up period. The TTS ranged from 18 to 55 weeks with a median of 27 weeks (25th and 75th percentiles of 25 to 30 weeks respectively) (Fig. 1).

**Fig. 2:**
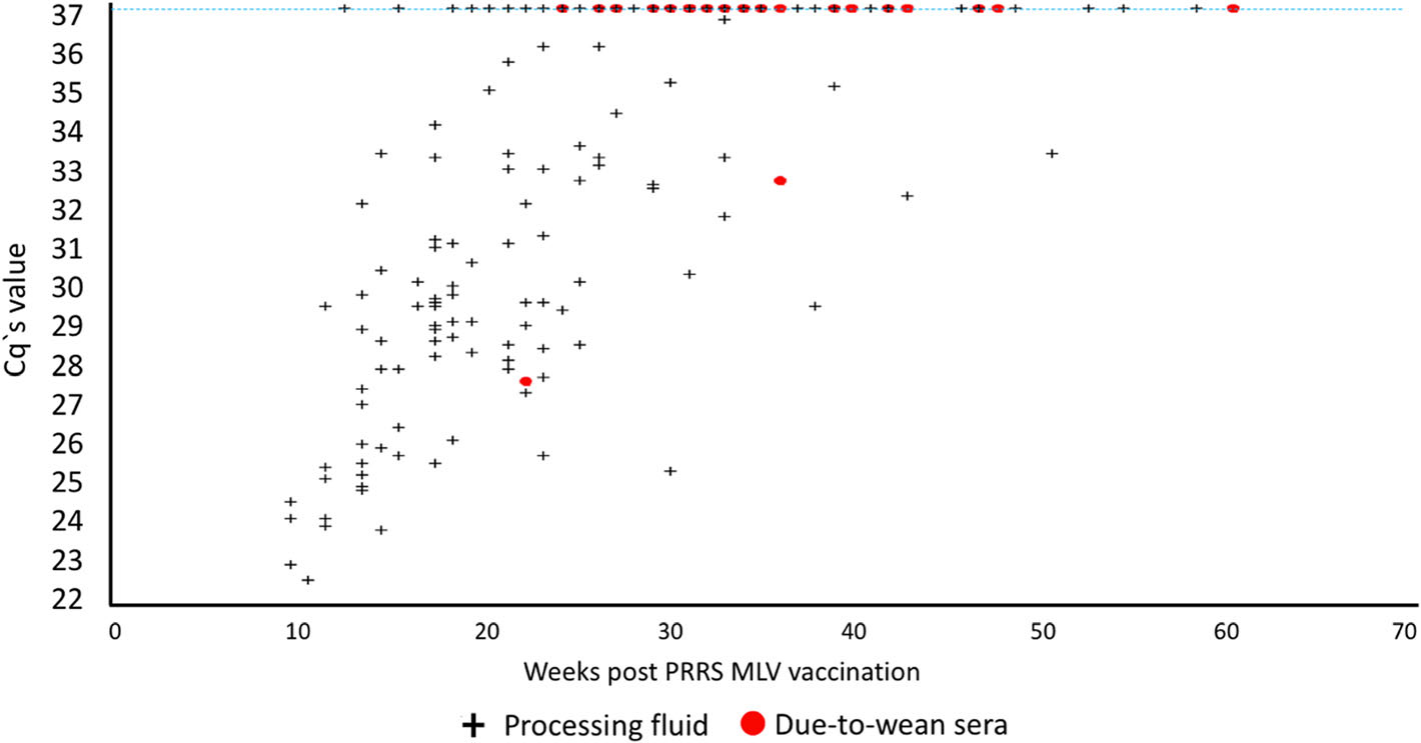
PRRSv RT-qPCR Cq values from processing fluids samples, from 10 to 61 weeks post vaccination. Green crosses represent processing fluids samples, and red dots represent due-to-wean piglet sera. The dotted blue line represents the cut-off for positive and negative. Cq value ≥37 are considered negative

Sera was collected at 30 different timepoints from 60 due-to-wean piglets across a total of 28 farms. The overall frequency to obtain negative results for PRRS by RT-qPCR in the first collection was 92.86% (26 of 28 farms), CI 83.32 to 100%. At least one PCR-positive result on due-to-wean sera was obtained in two farms: one at 22 weeks (farm A), and another at 36 weeks post MLV exposure (farm B). On farm A, further processing fluids testing at 23 and 27 weeks post-MLV had negative results for PRRSv by RT-qPCR. Subsequently, blood collected from due-to-wean piglets was tested at 29 weeks post-MLV with negative results also. On farm B, processing fluids taken on week 38 were PCR-positive, and at weeks 42 and 46 both were negative. The corresponding due-to-wean serum from that cohort of piglets tested negative for PRRSv by RT-qPCR. PRRSv type 1, also known as European, was not detected (Fig. 2).

At the end of this study, 26 farms had reintroduced PRRS-naïve gilts to the herd. Of those, 16 farms tested a range of 25 to 60 incoming gilts by ELISA. Collection of less than 60 sera samples took place when the entered number of gilts did not reach 60 individual gilts. From the 16 farms tested by ELISA 15 (93.76% CI 81.89 to 100%) had all samples testing negative by ELISA, while one farm with at least one sample testing positive. In the farm with positive ELISA results, there was only one seropositive gilt (S/P ratio 0.439). This same gilt was tested negative by RT-qPCR on serum sample. On that farm, results for PRRSv by RT-qPCR on processing fluids continued to test negative for the subsequent four batches of weaned pigs, and the subsequent batch of introduced gilts tested ELISA-negative after 30 days of introduction in the breeding herd. The two farms enrolled in this study that operated in continuous breeding-farrowing system obtained TTS at 20 and 27 weeks post MLV, respectively.

## Discussion

This study was a prospective study using processing fluids as an indicator of PRRSv presence at the time of piglet castration and tail docking (i.e. piglet processing). This is the first study describing the use of processing fluids as a screening method to monitor breeding herds for PRRSv over time.

The overall median TTS of 27 weeks agree with findings from previous field studies on time to produce PRRS-negative piglets at weaning following load-close-expose program using MLV vaccine [3, 9]. Two important differences should be pointed out in this study. The first is that a narrower confidence interval was obtained for median time in our study ranging from 25 to 30 weeks. This relatively narrow confidence interval represents consistency of the results. The second point is that this study was performed in a batch farrow system, and this could be a potential contributor for narrowing these results for a more consistent base. The removal of all weaning piglets from the farm between batches (i.e. all in-all out in the farrowing barn) could have potentially helped to reduce virus circulation in farrowing litters. Farms of this study, which implemented LCE with attenuated PRRS virus vaccine, reached TTS 5 weeks earlier (median time of 27 weeks) than a previous report in the literature (median time of 32 weeks) of farms using a similar strategy [3]. This shorten TTS may have had been influenced by the batch-farrowing system which produces an all in-all out piglet flow in the farrowing barns.

A total of 29 farms were enrolled in this study. There were thirty sera collection timepoints from the 28 farms that achieved TTS. In 92.86% of the farms (26 of 28) the results were negative in the first sera collection, which demonstrates that processing fluids was a great indicator of PRRSv circulation in suckling piglets. One farm had positive result on piglet sera at 22 weeks post MLV. Previous negative results for this farm were obtained via processing fluids at 15 and 19 weeks post MLV, respectively. As a comparison, all other farms with a negative result on processing fluid before 17 weeks failed to have a second negative result in the upcoming collection.

The methodology used to screen the newborn population for PRRSv with processing fluids, followed by testing the due-to-wean population with sera had 93.75% of the herds classified as stable for PRRSv, demonstrating an improvement for monitoring purposes when compared with previous work where only sera samples were used and 12.5% of the herds re-broke with the same PRRSv in less than 3 months after been declared stable [10]. Introduction of gilts occurred in 26 sow farms until the endpoint of the study, and 16 had gilt sera tested by ELISA. Ninety-four percent of farms (15 of 16) had ELISA-negative tests on incoming gilts, supporting that the PRRSv monitoring protocol used in this study (processing fluids testing followed up by due-to-wean piglet testing) was a good indicator of breeding herd PRRSv status. There was only one farm (1 of 16) that had ELISA-positive results, which was in one gilt with a low S/P ratio (0.439) and RT-qPCR-negative on the same serum sample. The farm continued to test negative by RT-qPCR on processing fluids over time, and the subsequent incoming gilt batch tested negative on ELISA. Altogether, results support that the herd was truly stable in terms of virus shedding. Additionally, none of the 16 farms that achieved TTS and tested gilt sera negative by ELISA reported another PRRSv outbreak until the end of this study.

There was an overall increase of RT-qPCR Cq values over time. This information is valuable to help practitioners to align expectation regarding remaining time (in weeks) to produce PRRSv-negative piglets at processing time.

Processing fluid was here demonstrated as valid tool be used to screen breeding herds undergoing PRRSv elimination. The AASV criteria relies on testing due-to-wean piglet population to classify a herd as stable (Category II) for PRRSv. In this work only 2 farms had one event each were sera collection in due-to wean piglets’ population resulted in positive results for PRRSv after two consecutive negative results (i.e. two 4-week batches) on processing fluid were previous obtained. As a reminder in a four week-batch system, two sampling weeks is equivalent to 8 continuous weeks. These results suggest that the determination of herd stability should not rely solely on screening the newborn population by using processing fluid-based testing. Before considering a herd as stable for PRRSv, a testing on due-to-wean piglet population is encouraged using a reliable sample type for this age category such as individual sampling piglets with sera [2] and or by family-oral-fluids [11], a population based sampling method. In summary, a suggested schema for considering a herd stable using processing fluid to screen for PRRSv should consider at least 8 consecutive negative weeks with negative results on processing fluid followed by a test in the due-to-wean pig population. Additionally, monitoring herd-level parameters such as increasing preweaning mortality, number of weekly abortion and weekly sow mortality can give additional information of disease activity and PRRSV should be investigated targeting those animals for testing.

## Conclusion

Processing fluids are a practical sample type to be considered for PRRSv monitoring in breeding herds undergoing virus elimination. In this study, 93% of farms that reached two consecutive negative results for PRRSv by RT-qPCR also had PRRS RT-qPCR -negative results on serum at weaning on the same cohort of pigs. From those 28 farms that achieved TTS, 16 had gilt tested by ELISA and 94% had incoming gilts testing negative on ELISA after 30 days of introduction in the breeding herd.

## Data Availability

Restrictions apply to the availability of individual diagnostic result, and sow farm information due producer confidentiality, and are not publicly available.

## References

[1] Holtkamp DJ, Kliebenstein JB, Neumann EJ, Zimmerman JJ, Rotto HF, Yoder TK, Wang C, Yeske PE, Mowrer CL, Haley CA (2013). Assessment of the economic impact of porcine reproductive and respiratory syndrome virus on United States pork producers. J Swine Health Prod.

[2] Holtkamp DJ, Polson DD, Torremorell M (2011). Terminology for classifying swine herds by porcine reproductive and respiratory syndrome virus status. J Swine Health Prod.

[3] Linhares DC, Cano JP, Torremorell M, Morrison RB (2014). Comparison of time to PRRSv-stability and production losses between two exposure programs to control PRRSv in sow herds. Prev Vet Med.

[4] Kittawornrat A, Panyasing Y, Goodell C, Wang C, Gauger P, Harmon K, Rauh R, Desfresne L, Levis I, Zimmerman J (2014). Porcine reproductive and respiratory syndrome virus (PRRSV) surveillance using pre-weaning oral fluid samples detects circulation of wild-type PRRSV. Vet Microbiol.

[5] Graham J, Rademacher C, Swalla R (2013). Use of oral fluid sampling in suckling pigs for PRRSV monitoring.

[6] Lopez WA, Angulo J, Zimmerman JJ, Linhares DCL (2018). Porcine reproductive and respiratory syndrome monitoring in breeding herds using processing fluids. J Swine Health Prod.

[7] Lopez, W.; Linhares, D. Processing fluids, blood serum, and tail blood swabs to detect PRRSV RNA and PCV2 DNA by PCR-Based assays, 2017 ISU James D. McKean Swine Disease Conference, Ames, IA, Ames, IA, 2017; p 69.

[8] Lopez WA, Zimmerman JJ, Angulo J, Linhares DCL. Processing fluids for detection of PRRS activity in neonates, 2017 ISU James D, vol. 2017. Ames, IA, Ames, IA: McKean Swine Disease Conference. p. 65.

[9] Betlach C, Linhares DCL, Anderson A, Morrison R (2016). Evaluation of time to stability and associated risk factors in sow herds infected with PRRS 1–7-4.

[10] Linhares DCL. Evaluation of immune management strategies to control and eliminate porcine reproductive and respiratory syndrome virus (PRRSv): University of Minnesota; 2013.

[11] Almeida M, Allison G, Silva GS, Holtkamp D, Zimmerman J, Linhares D (2018). Field-Based Studies on PRRSv, ISU James D.

